# A Computational Approach for Mapping Heme Biology in the Context of Hemolytic Disorders

**DOI:** 10.3389/fbioe.2020.00074

**Published:** 2020-03-06

**Authors:** Farah Humayun, Daniel Domingo-Fernández, Ajay Abisheck Paul George, Marie-Thérèse Hopp, Benjamin F. Syllwasschy, Milena S. Detzel, Charles Tapley Hoyt, Martin Hofmann-Apitius, Diana Imhof

**Affiliations:** ^1^Pharmaceutical Biochemistry and Bioanalytics, Pharmaceutical Institute, University of Bonn, Bonn, Germany; ^2^Department of Bioinformatics, Fraunhofer Institute for Algorithms and Scientific Computing (SCAI), Sankt Augustin, Germany

**Keywords:** heme, hemolytic disorders, signaling pathways, knowledge graphs, biological expression language

## Abstract

Heme is an iron ion-containing molecule found within hemoproteins such as hemoglobin and cytochromes that participates in diverse biological processes. Although excessive heme has been implicated in several diseases including malaria, sepsis, ischemia-reperfusion, and disseminated intravascular coagulation, little is known about its regulatory and signaling functions. Furthermore, the limited understanding of heme’s role in regulatory and signaling functions is in part due to the lack of curated pathway resources for heme cell biology. Here, we present two resources aimed to exploit this unexplored information to model heme biology. The first resource is a terminology covering heme-specific terms not yet included in standard controlled vocabularies. Using this terminology, we curated and modeled the second resource, a mechanistic knowledge graph representing the heme’s interactome based on a corpus of 46 scientific articles. Finally, we demonstrated the utility of these resources by investigating the role of heme in the Toll-like receptor signaling pathway. Our analysis proposed a series of crosstalk events that could explain the role of heme in activating the TLR4 signaling pathway. In summary, the presented work opens the door to the scientific community for exploring the published knowledge on heme biology.

## Introduction

Heme is an iron ion-coordinating porphyrin derivative essential to aerobic organisms (Zhang, 2011). It plays a crucial role as a prosthetic group in hemoproteins involved in several biological processes such as electron transport, oxygen transfer, and catalysis (Smith and Warren, 2009; Zhang, 2011; Kühl and Imhof, 2014; Poulos, 2014). Besides its indispensable role in hemoproteins, it can act as a damage-associated molecular pattern leading to oxidative injury, inflammation, and consequently, organ dysfunction (Jeney, 2002; Wagener et al., 2003; Dutra and Bozza, 2014). Plasma scavengers such as haptoglobin and hemopexin bind hemoglobin and heme, respectively, thus keeping the concentration of labile heme at low concentrations (Smith and McCulloh, 2015). However, at high concentrations of hemoglobin and, consequently heme, these scavenging proteins get saturated, resulting in the accumulation of biologically available heme (Soares and Bozza, 2016). With respect to hemolytic diseases, the formation of labile heme at harmful concentrations has been a subject of research for some years now (Roumenina et al., 2016; Soares and Bozza, 2016; Gouveia et al., 2017).

Biomedical literature is an immense source of heterogeneous data that are dispersed throughout hundreds of journals. Furthermore, the majority of the results are scattered and published as unstructured free-text, or at best, presented in tables and cartoons representing the experimental study or biological processes and pathways. These shortcomings, combined with the exponential growth of biomedical literature, prevent the healthcare community and individual researchers from being aware of all the available information and knowledge in the literature. With the introduction of new technologies and experimental techniques, researchers have made significant advances in heme-related research and its role in the pathogenesis of numerous hemolytic diseases such as sepsis (Larsen et al., 2010; Effenberger-Neidnicht and Hartmann, 2018), malaria (Ferreira et al., 2008; Dey et al., 2012), and β-thalassemia (Vinchi et al., 2013; Conran, 2014; Garcia-Santos et al., 2017). In these diseases, large amounts of heme are released from ruptured erythrocytes and can potentially wreak havoc (Tolosano et al., 2010). Thus, it is crucial to develop new strategies that capture and exploit the vast amount of literature knowledge surrounding heme to better understand its mechanistic role in hemolytic disorders.

Biological knowledge formalized as a network can be used by clinicians as research and information retrieval tools, by biologists to propose *in vitro* and *in vivo* experiments, and by bioinformaticians to analyze high throughput *-omics* experiments (Catlett et al., 2013; Ali et al., 2019). Further, they can be readily semantically integrated with databases and other systems biology resources to improve their ability to accomplish each of these tasks (Hoyt et al., 2018). However, enabling this semantic integration requires organizing and formalizing the knowledge using specific vocabularies and ontologies. Although this endeavor involves significant curation efforts, it is key to the success of the subsequent modeling steps. Therefore, in practice, knowledge-based disease modeling approaches have been conducted only for major disorders such as cancer (Kuperstein et al., 2015) or neurodegenerative disorders (Mizuno et al., 2012; Fujita et al., 2014). In summary, while the scarcity of mechanistic information and the necessary amount of curation often impede launching the aforementioned approaches, modeling and mining literature knowledge provide a holistic picture of the field of interest. Furthermore, the underlying models derived from such approaches have a broad range of applications including hypothesis generation, predictive modeling and drug discovery.

Here, we present two resources aimed at assembling mechanistic knowledge surrounding the metabolism, biological functions, and pathology of heme in the context of selected hemolytic disorders. The first resource is a terminology formalizing heme-specific terms that have until now not been covered by other standard controlled vocabularies. The second resource is a heme knowledge graph (HemeKG), that is, a network comprising more than 700 nodes and more than 3,000 interactions. It was generated from 46 selected articles as the first attempt of modeling the knowledge, which is available from more than 20,000 heme-related publications. Finally, we demonstrate both resources by analyzing the crosstalk between heme biology and the TLR4 signaling pathway. The results of this analysis suggest that the activation profile for labile heme as an extracellular signaling molecule through TLR4 induces cytokine and chemokine production. However, the underlying molecular mechanism and individual pathway effectors are not fully understood and need further exploration.

## Materials and Methods

**FIGURE 1:**
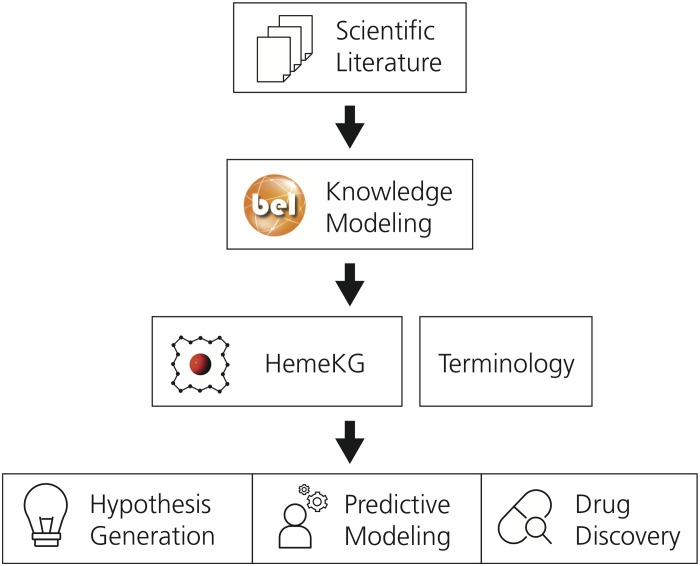
The workflow used to generate the supporting terminology and HemeKG. The first step involves the selection of relevant scientific literature. Next, evidence from this selected corpus is extracted and translated into BEL to generate a computable knowledge assembly model, HemeKG. In parallel to the modeling task, a terminology to support knowledge extraction of articles about the heme molecule was built. Finally, HemeKG can be used for numerous tasks such as hypothesis generation, predictive modeling and drug discovery.

This section describes the methodology used to generate the mechanistic knowledge graph and its supporting terminology. Subsequently, it outlines the approach followed to conduct the pathway crosstalk analysis. A schematic diagram of the methodology is presented in Figure 1.

### Knowledge Modeling

In order to identify recently published articles (i.e., published in the last 10 years) describing the role of heme in hemolytic disorders, PubMed was queried with the following: (“heme” AND “hemolysis”) OR (“heme” AND “thrombosis”) OR (“heme” AND “inflammation”) AND (“2009”[Date – Publication]: “3000”[Date – Publication]). The resulting 3,108 articles were manually filtered by removing articles that were deemed too general or lacked a biochemical focus, as judged by expert opinion. After this filtering step, 6 reviews and 40 original research articles were selected for knowledge extraction and modeling. Knowledge was manually extracted and curated from this selected corpus using the official Biological Expression Language (BEL) curation guidelines from http://openbel.org/language/version_2.0/bel_specification_version_2.0.html and http://language.bel.bio as well as additional guidelines from https://github.com/pharmacome/curation.

Evidence from the selected corpus was manually translated into BEL statements together with their contextual information (e.g., cell type, tissue and dosage information). For instance, the evidence “Heme/iron-mediated oxidative modification of LDL can cause endothelial cytotoxicity and – at sublethal doses – the expression of stress-response genes” (Nagy et al., 2010) corresponds to the following BEL statement:

SET Cell = “endothelial cell”
a(CHEBI:“oxidised LDL”) pos bp(MESH:“Cytotoxicity, Immunologic”).

### Generation of a Supporting Terminology

During curation, a terminology was generated to support the standardization of domain-specific terminology encountered during the curation of articles related to the heme molecule. The aim of the terminology is to catalog and harmonize terms not present in other controlled vocabularies such as ChEBI (Degtyarenko et al., 2007) for chemicals, or Gene Ontology [GO; (Ashburner et al., 2000)] and Medical Subject Headings [MeSH; (Rogers, 1963)] for pathologies. Thus, each term was checked by two experts in the field assisted by the Ontology Lookup Service [OLS; (Cote et al., 2010)] to avoid duplicates with other terminologies or ontologies. Furthermore, we required that each entry included the following metadata: an identifier, a label, a definition, an example of usage in a sentence, and references to articles in which it was described. Furthermore, a list of synonyms was also curated in a separate file to facilitate the use of the terminology in annotation or text mining tasks. The supporting terminology is included in the Supplementary Material and can also be found at https://github.com/hemekg/terminology.

### Analyzing Pathway Crosstalk Between Heme and the Toll-Like Receptor Signaling Pathway

Crosstalk analysis aims to study how two or more pathways communicate or influence each other. While there exist, numerous methodologies designed to investigate pathway crosstalk, the majority of these approaches exclusively quantify such crosstalk based on the overlap between a pair of pathways without delving into the nature of the crosstalk (Donato et al., 2013). In this section, we demonstrate how combining knowledge from HemeKG with a canonical pathway reveals mechanistic insights on the crosstalk between two different pathways.

Because of the amount of effort required to manually analyze crosstalk across multiple pathways, we conducted a pathway enrichment analysis on three pathway databases [i.e., KEGG Kanehisa et al., 2016), Reactome (Fabregat et al., 2017), WikiPathways (Slenter et al., 2017)] to identify pathways enriched with the gene set extracted from the entire Heme knowledge map. The enrichment analysis evaluated the overrepresentation of the genes present in HemeKG for each of the pathways in the three aforementioned databases using Fisher’s exact test (Fisher, 1992). Furthermore, Benjamini–Yekutieli method under dependency was applied to correct for multiple testing (Yekutieli and Benjamini, 2001). Manual inspection of the enrichment analysis results revealed that the Toll-like receptor (TLR) signaling pathway was the most enriched pathway in Reactome and WikiPathways, and the third most enriched in KEGG (Supplementary Table S1). Therefore, this pathway was selected for study in the subsequent investigation.

First, the three different representations of this pathway were downloaded from each database and converted to BEL using PathMe (Domingo-Fernández et al., 2019). Next, the three BEL networks were combined with the HemeKG network highlighting their overlaps (Supplementary Figures S1, S2) in order to specifically analyze these parts of the combined network. Finally, five experts in the field reconstructed the hypothesized pathways from the combined network. The hypothesized pathways were depicted following the guidelines for scientific communication of biological networks outlined by Marai et al. (2019).

## Results

### Building a Mechanistic Knowledge Graph Around Heme Biology in the Context of Hemolytic Disorders

**FIGURE 2:**
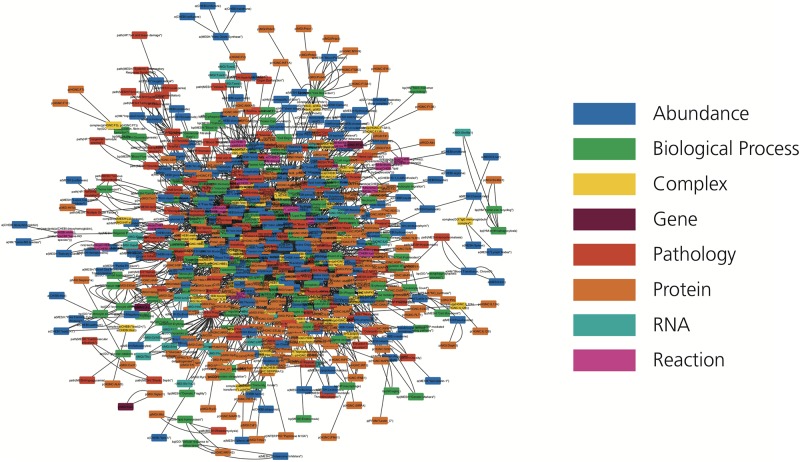
The HemeKG network. Nodes are colored by their different functions in BEL (see legend).

We introduce the first knowledge graph made publicly available to the biomedical and bioinformatics community focused on heme biology (Figure 2). The presented heme knowledge graph was based on the selection of 40 original research articles and 6 review articles related to heme and its role in several pathways. These pathways include the tumor necrosis factor (TNF) and nuclear factor κ-light-chain-enhancer of activated B cells (NF-κB) signaling pathways, and the complement and coagulation cascades, through which heme plays a role in hemolysis, inflammation and thrombosis (Dutra and Bozza, 2014; L’Acqua and Hod, 2014; Roumenina et al., 2016; Martins and Knapp, 2018; Vogel and Thein, 2018). The focus of the review articles was chosen because of the relevance of these diseases and complications to large numbers of patients (L’Acqua and Hod, 2014; Litvinov and Weisel, 2016; Roumenina et al., 2016; Effenberger-Neidnicht and Hartmann, 2018). All of these pathologies are known to be interconnected and mapping them in relation to heme is promising for the discovery of yet overlooked links.

Following the guidelines outlined in the Methods section, knowledge was manually extracted and encoded from each of these 46 articles using BEL because of its ability to represent not only causal, but also correlative and associative relationships found in the literature, as well as corresponding provenance and experimental contextual information. This curation exercise resulted in HemeKG, a knowledge graph containing 775 nodes (Table 1) and 3,051 relations (Table 2), as well as contextual information ranging from cellular and anatomical localization to different states of the heme molecule (Supplementary Figure S1). Annotations, such as time point and concentration, enabled us to capture time dependencies between entities. By using this contextual information and the multiple biological scales presented in the model, we have not only been able to represent a part of heme’s interactome (Figure 2), but also established several links to phenotypes and clinical endpoints. Both represent essential considerations for the design of future clinical studies of hemolytic conditions.

**TABLE 1:** Summary of unique nodes for each entity class.

**Abundances**	**Genes**	**RNAs**	**Proteins**	**Complexes**	**Reactions**	**Pathologies**	**Biological processes**	**Total**
200	4	25	226	54	17	128	121	**775**

*Each entity class corresponds to the terms formalized in BEL (more information at https://language.bel.bio).*

**TABLE 2:** Summary of relationship classes.

**Increase**	**Decrease**	**Positive correlation**	**Negative correlation**	**Has component**	**Association**	**Causes No change**	**Ontological relations**	**Total**
639	380	1,322	440	113	54	39	64	**3,051**

*Each class corresponds to the relationships formalized in BEL (more information at https://language.bel.bio). The ontological relations class includes the following relationships: has reactant, has product, and has variant.*

Finally, to facilitate the use of the curated content in this work, BEL documents are bundled with a dedicated Python package that enables direct access to the content, provides conversion utilities and allows for network exploration. Both the BEL documents and the Python package are available at https://github.com/hemekg/hemekg.

### Curating a Supporting Heme Terminology

The specificity of our work, together with the lack of contextual terminologies related to heme biology, prompted us to generate a supporting terminology focused on heme. It contains more than 50 terms that delineate heme-related entities, such as biological processes, proteins, or pathologies that are not yet included in other standard resources such as (GO Ashburner et al., 2000). Building this terminology not only allowed us to describe entities with more expressiveness, but also facilitates text mining or annotation tasks related to the heme molecule in the future. The terminology is available at https://github.com/hemekg/terminology.

### Dissection of the Crosstalk Between Heme and TLR Using HemeKG

The established heme knowledge graph can be used to study the crosstalk of heme biology with a pathway of interest. HemeKG is of special interest in the context of hemolytic disorders, such as malaria and sickle cell anemia, because these diseases are associated with the release of heme into circulation. Heme can then exert a detrimental role by regulating several proteins and signaling pathways (Kühl and Imhof, 2014). In order to select a pathway that highly overlaps with the generated network, we conducted pathway enrichment analysis using three major databases (i.e., KEGG, Kanehisa et al., 2016), Reactome (Fabregat et al., 2017), and WikiPathways (Slenter et al., 2017). The results of the enrichment analysis in the three databases pointed to TLR signaling as the most enriched pathway (Supplementary Table S1). Thus, we proceeded to analyze the crosstalk between this pathway and heme biology by exploring the overlap between HemeKG and the TLR pathways in the three aforementioned databases. Although heme has been linked to numerous (TLRs) including TLR2, TLR3, TLR4, TLR7, and TLR9 (Figueiredo et al., 2007; Lin et al., 2010; Dutra and Bozza, 2014; Min et al., 2017; Merle et al., 2019; Sudan et al., 2019), our analysis was prioritized on the most well-documented interaction, the one between heme and TLR4. Heme stimulates TLR4 to activate NF-κB secretion via myeloid differentiation primary response 88 (MyD88)-mediated activation of lκB (IKK) (see below). Activated IKK promotes the proteolytic degradation of NFKBIA. The phosphorylated IKK complex indirectly activates NF-κB and mitogen-activated protein kinases, such as JNK (C-Jun N-terminal kinase), ERK, and p38 leading to the secretion of TNF-α, interleukin 6 (IL6), IL1B, and keratinocyte-derived chemokine (Dutra and Bozza, 2014). This finally results in an activation of the innate immunity and the generation of proinflammatory factors, which reflects the relevance of heme in several disorders comprising inflammation and infection.

**FIGURE 3:**
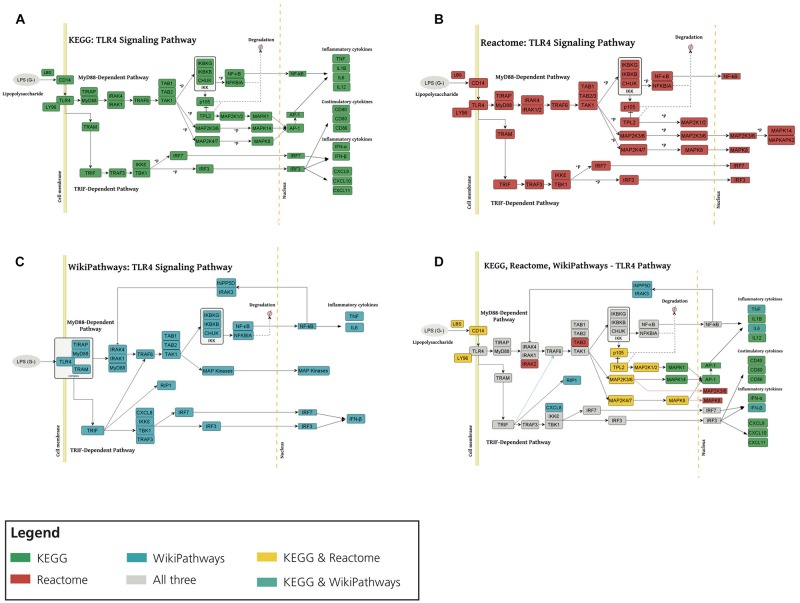
Consensus around the TLR4 signaling pathway in three major pathway databases. TLR4 signaling pathway visualization of KEGG **(A)**, Reactome **(B)**, and WikiPathways **(C)**. **(D)** Superimposing TLR4 signaling pathway from KEGG, Reactome and WikiPathways. Each color corresponds to the presence of the given node in one or multiple databases (see Legend). MyD88, TAK1, IKK complex, MAP kinases, TNF, NF-κB, TRIF and IRF3 emerged in all three databases and also in HemeKG. KEGG and Reactome showed identical representations of the TLR4 pathway whereas WikiPathways was different in a way that nuclear NF-κB activates INPP5D-IRAK3 (inositol polyphosphate-5-phosphatase D IL1 receptor associated kinase 3) complex which inhibits the activity of IRAK1/IRAK4 (IL1 receptor associated kinase 1/4).

We first investigated the consensus of the three different representations of the TLR4 signaling pathway (Figure 3A). We observed that, overall, all three representations share a high degree of consensus as illustrated in Figures 3B–D. Here, we would like to point out that while KEGG and Reactome present practically identical representations, the WikiPathways representation exhibits slight differences. These differences and complementarities between pathways provide us with a more comprehensive view of the studied pathways, as illustrated by our previous work (Domingo-Fernández et al., 2019).

**FIGURE 4:**
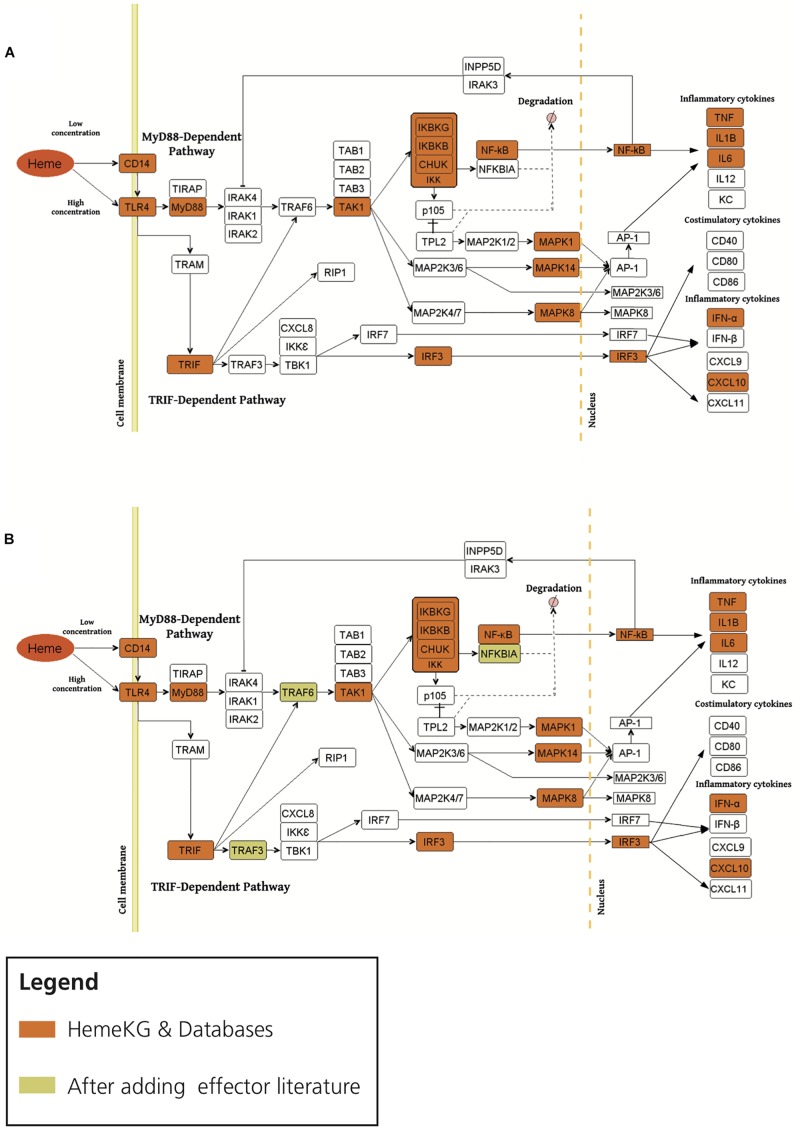
Overlaying the consensus TLR4 signaling pathway in databases with HemeKG (**A:** original overlaid network, **B:** overlaid network after inclusion of literature evidence for effectors). The orange colored boxes display the common effector molecules between the canonical TLR4 signaling pathway and induced TLR4 signaling pathway stimulated by labile heme. Heme/TLR4 activates the adaptor molecule MyD88. Activated MyD88 promotes the degradation of NFKBIA (NF-κB inhibitor α) through phosphorylation of the IKK complex (inhibitor of nuclear factor κB kinase complex), thus promoting NF-κB (nuclear factor κ-light-chain-enhancer of activated B cells) and MAPKs (mitogen-activated protein kinases) stimulation leading to the secretion of TNF-α, IL6, IL1B and KC (keratinocyte-derived chemokine) (Fortes et al., 2012; Dutra and Bozza, 2014). The TRIF (Toll-like receptor adaptor molecule 1) dependent pathway is activated upon signaling of heme through TLR4 leading to the activation of IRF3 (interferon regulatory factor 3) stimulating the secretion of interferons (i.e., IFN-α) and CXCL10 (C-X-C motif chemokine ligand 10) (Dickinson-Copeland et al., 2015). However, the activation profiles for IRAK1/2, TRAF6, TRAM, TRAF3, TBK1/IKK epsilon complex and IRF7 are not yet studied for heme-TLR4 signaling pathway.

Second, in order to study the overlap between TLR4 signaling pathway and heme biology, we overlaid the consensus network of the pathway with HemeKG (Figure 4). Superimposing both networks revealed that MyD88, TAK1, IKK complex, MAP kinases, TNF, NF-κB, Toll-like receptor adaptor molecule 1 (TRIF), and interferon regulatory factor 3 (IRF3) were present in all three databases and in our model. However, several effector molecules, which were found in the three databases, were not found in our heme knowledge graph (HemeKG), for example, IL1 receptor-associated kinase proteins 1, 2, and 4 (IRAK1, IRAK2, and IRAK4, respectively); TNF receptor-associated factor 6 (TRAF6); TAB1-3; and others (Figure 4A). Thus, we specifically searched for literature reports of these effectors in the context of heme signaling, by entering the respective queries in PubMed, as this knowledge might not have been sufficiently covered by the 40 original research articles selected to establish HemeKG.

The activation profile for labile heme as an extracellular signaling molecule through TLR4 was suggested to be similar to the one established via Lipopolysaccharides (LPS) as signaling molecule from standard pathway databases (Pålsson-Mcdermott and O’Neill, 2004). This pathway begins with the induction of TIRAP (Mal)-associated MyD88 signaling on the one hand (Horng et al., 2002), and TRAM (TICAM-2)-associated TRIF (TICAM-1)-signaling, on the other hand (Seya et al., 2005), resulting in the upregulation of proinflammatory cytokines and chemokines (Figure 4). MyD88 protein as an adaptor has been shown to interact with IL1 receptor-associated kinase (IRAK) proteins 1, 2, and 4 to start the signaling cascade involving TRAF6, which is known to activate IKK in response to proinflammatory cytokines. However, in our heme knowledge graph the connections between IRAKs, TRAF6, and TAB proteins were missing (Figure 4A). By taking a closer look at these effectors in the context of heme, we found various information for example TRAF6 indicating both a direct and indirect link to heme-induced signaling via TLRs (Hama et al., 2012; IJssennagger et al., 2012; Park et al., 2014; Huang et al., 2015; Meng et al., 2017). In contrast, other effector molecules such as IRAK and TAB proteins (Figure 4) were not described in heme signaling so far. We then performed a PubMed search for these missing terms in combination with “heme.” These findings led us to refine HemeKG so that only those signaling components for which no evidence was found manually still remain as white spots on the map (Figure 4B).

In addition, the preceding discussion has excluded parameters such as the concentration of labile heme available in the respective environment. This aspect will be particularly important, if heme-triggered signaling pathways are dependent on, or determined by the concentration of heme. At lower concentrations of heme, TLR4 signaling has been described to be CD14 dependent, whereas at high concentrations of heme, TLR4 activation does not require CD14 (Piazza et al., 2010; Figure 4missing). Also, there is a need to further investigate whether heme/TLR4 induction of the adapter molecule MyD88 is dependent or independent of TIRAP activation, similar to the LPS/TLR4 induced TIRAP-associated MyD88 signaling pathway. Furthermore, heme/TLR4 activates a pathway that leads to the activation of IRF3, resulting in the production of interferons for example, IFN-α (Dutra and Bozza, 2014) and overproduction of C-X-C motif chemokine 10 (CXCL10) (Lin et al., 2012; Dickinson-Copeland et al., 2015). In the literature, the molecular mechanism by which heme/TLR4-induced TRAF3 and IRF3/7 activation leads to the secretion of IFN-α and CXCL10 is not represented. It is therefore shown as a white box in the map (Figure 4B). Finally, the introduction of noncanonical pathways and receptor crosstalk-triggered cascades go beyond the scope of this work, but represent opportunities for future studies on heme signaling.

## Discussion

We have presented HemeKG, the first mechanistic model in the context of heme biology, as a viable solution to comprehensively summarize heme-related processes by bringing knowledge from disparate literature together. Furthermore, we have demonstrated how combining the knowledge from the heme knowledge graph with information available in pathway databases provides new insights into the network of interactions that regulate heme pathophysiology.

Because HemeKG was curated using standard vocabularies, its content can be linked to the majority of public databases. Therefore, enriching the HemeKG network with external data or incorporating its integrated knowledge into other resources is feasible. For example, the entire Bio2BEL framework^1^ can be used to scale up this resource by enriching HemeKG with dozens of widely used biomedical databases. In order to make HemeKG accessible to a wider audience, we uploaded it to BEL Commons - a web application for curating, validating, and exploring knowledge assemblies encoded in BEL (Hoyt et al., 2018). Users can interactively explore the network, make modifications, integrate additional resources via Bio2BEL, and share those modifications using its versioning system. Furthermore, the variety of formats that our resource can be converted to also facilitates its use by other systems biology tools such as Cytoscape (Shannon, 2003) and NDEx (Pratt et al., 2015). In summary, the characteristics of HemeKG make this resource suitable not only for hypothesis generation as presented in our case scenario, but also for clinical decision support as previously demonstrated with other systems biology maps (Ostaszewski et al., 2018). For instance, computational mechanistic models are currently being used in combination with artificial intelligence methods for a variety of predictive applications (Khanna et al., 2018; Esteban-Medina et al., 2019; Çubuk et al., 2019). Instead of contextless canonical pathways as until now (i.e., pathways describing normal physiology), HemeKG could be used for predicting drug response and for drug repurposing in numerous related disorders such as malaria and sepsis. Finally, the supporting terminology built during this work could be used for a broad range of applications from data harmonization to natural language processing.

A potential limitation of this study is that it is constrained to a specific literature corpus as we are aware that the presented knowledge graph captures only a part of a much larger interaction network. This tends to be a common challenge when constructing contextualized maps and is further compounded by the difficulty in assessing the coverage of a network, explaining why some nodes are missing in HemeKG compared to the three pathway databases used in this study. Furthermore, the bias in the scientific community against publishing negative results must also be acknowledged. A clear example is how the hypotheses of our crosstalk analysis could be complemented by this knowledge gap that could reveal new interesting hypotheses. Thus, future updates in HemeKG, as in any work of this kind, will be required while prioritizing time and effort (Rodriguez-Esteban, 2015). Further, advanced network-based analyses (Catlett et al., 2013) could be used to rank heme-related pathways in the context of a given *-omics* data set.

Although numerous interactions between heme and TLRs have been described in the literature (Lin et al., 2010; Min et al., 2017), their downstream effects have not been contextualized (i.e., presented in a coherent/integrated manner like a knowledge model does). The analysis we have presented focusing on the crosstalk between heme biology and the TLR signaling pathway has shed some light on how this crosstalk could be related to heme biology. However, there also exist other well-known pathways related to heme, that could be investigated by conducting similar analyses in the future.

## Data Availability Statement

The data sets and scripts of this study can be found at https://github.com/hemekg.

## Author Contributions

DI, MH-A, and DD-F conceived and designed the study. FH curated the data and conducted the main analysis supervised by AP, DI, and DD-F. M-TH, BS, MD, and AP assisted in selecting the corpora and interpreting the results. CH designed the curation guidelines and implemented the Python package. DD-F, FH, CH, M-TH, BS, MD, and DI wrote and reviewed the manuscript.

## Conflict of Interest

The authors declare that the research was conducted in the absence of any commercial or financial relationships that could be construed as a potential conflict of interest.

## Footnotes

1 https://github.com/bio2bel
